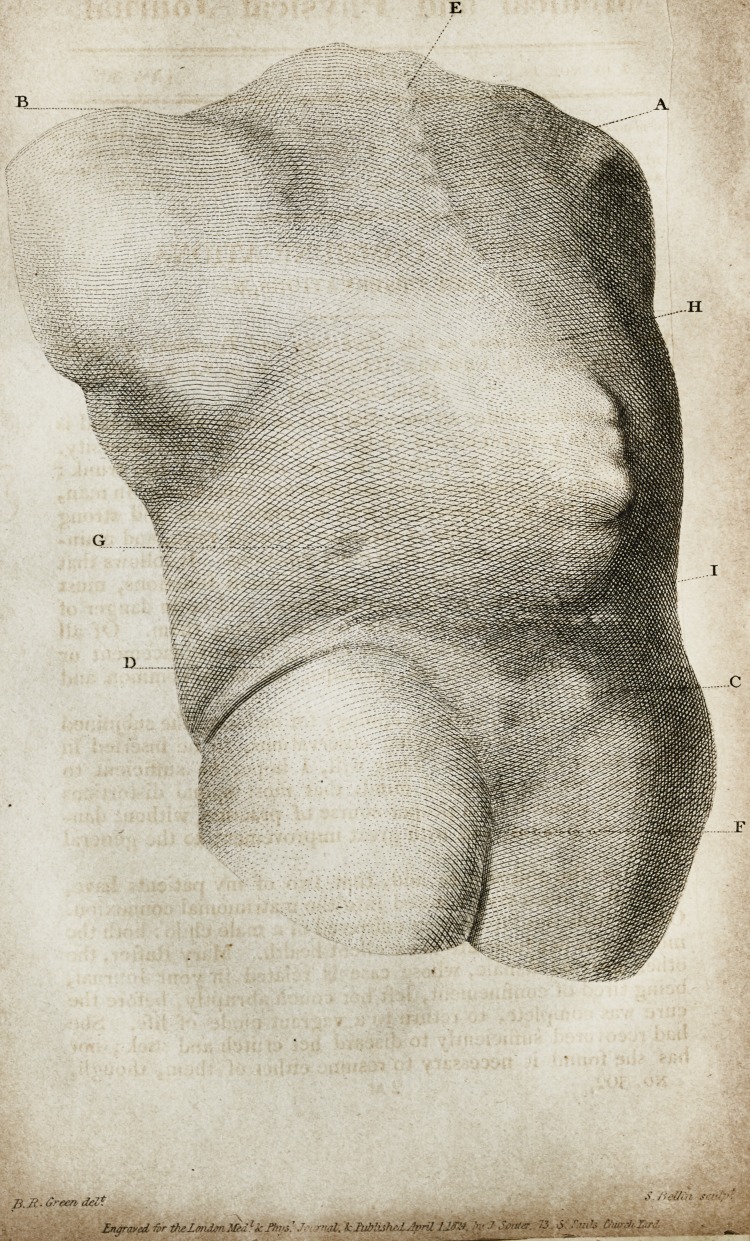# Observations on the Pathology and Treatment of Spinal Diseases

**Published:** 1824-04

**Authors:** Edward Harrison


THE LONDON

### Medical and Physical Journal.

4 OF VOL. LI.] APRIL, 1824. [No. 302.

For many fortunate discoveries in medicine, and for the detection of numerous errors, the world is indebted to the rapid circulation of Monthly Journals; and there never existed any work, to which the Faculty, in Europe and America, were under deeper obligations, than to the Medical and Physical Journal of London, now forming a long, but an invaluable, series.—RUSH.

### ORIGINAL COMMUNICATIONS,
### SELECT OBSERVATIONS, &c.

ART. I.—*Observations on the Pathology and Treatment of Spinal Diseases.* By EDWARD HARRISON, M.D. F.R.A.S. ED.

[With Engravings.]

THE spine constitutes an essential part of many animals, and is destined to perform offices of great importance and diversity. It must be flexible, to provide for the motions of the trunk; and strong, to protect the inclosed nervous substance. In man, the spinal column is provided with separate bones and strong ligaments, to enable him at pleasure to bend, raise, and maintain himself in the erect and in other positions. It follows that an organ, which is adapted to many different functions, must consist of numerous and distinct materials, and be in danger of getting frequently out of order by employing them. Of all injuries to the spine, distortions arising from displacement or luxation of its vertebræ are, perhaps, the most common and distressing.

I shall, therefore, offer no apology for inclosing the subjoined cases, with the accompanying observations, to be inserted in your valuable Journal. They will, I hope, be sufficient to convince all unprejudiced minds that most spinal distortions admit of removal by a proper course of practice, without danger to the patient, and with great improvement to the general health.

I have the pleasure to add, that two of my patients have, since their recovery, entered into the matrimonial connexion. One of them has lately been delivered of a male child: both the mother and her baby are in excellent health. Mary Rafter, the other married female, whose case is related in your Journal, being tired of confinement, left her couch abruptly, before the cure was complete, to return to a vagrant mode of life. She had recovered sufficiently to discard her crutch and stick; nor has she found it necessary to resume either of them, though, since the treatment was discontinued, she has till lately wandered about the country with haberdashery articles. This laborious employment exposes her to great danger of a relapse, by the weight of the goods which she has to carry. Hitherto she has continued well, though she has sometimes walked more than thirty miles in the day.

The case of the married lady makes the seventh in my list, published in your Journal. As the truth of many circumstances related in it have been more than suspected by certain members of the faculty, who have no personal knowledge of the patient, nor other proper means of judging for themselves, I propose to enter more into detail than formerly, for the satisfaction of your readers. This lady convinced herself, by feeling with her fingers along the spine, and viewing it in a looking-glass, that the whole column had become rugged and uneven, when I was consulted: it is now, she says, smooth and regular. Nor are the proofs of displacement and restoration confined to the patient and myself: the family surgeon made a report to the same effect, before I was applied to. This gentleman, she says, was so forcibly struck with what he discovered, that he requested the lady with whom she then resided to be sent for. On her entering the apartment, he pointed out, and made her feel the vertebral eminences and depressions, as well in the neck and between the shoulders, as in the back and loins. Having satisfied their minds on these points, Mrs. F. became my patient, and continued under my care till she was cured. After I had taken my leave, the same practitioner was, it seems, desired to examine her again, in the presence of the lady above referred to. The spine was by both declared to be completely reinstated, the projecting and irregular vertebræ being no longer distinguishable from the rest. The patient has been visited, at my desire, by several different practitioners, who interrogated her particularly about the facts recorded in the case; they also carefully examined the spinal column. To them and to her, therefore, I shall not hesitate to refer, on a proper application being made to me, for the truth of the above statement, as well as for all that I have related on the same subject in a former Number of your Journal. These gentlemen likewise accompanied me to several other spinal sufferers, and will, I am sure, be ready to state what they think of my practice.

Having, I trust, convinced your readers that my former representation of Mrs. F.'s case is correctly given, I shall not stop to notice the vague and inconsistent rumours of prejudiced individuals, who have doubtless private reasons of their own for endeavouring to misrepresent my professional character.

*Holles-street, Cavendish-square;*

*Feb. 10th,* 1824.

### PART I.

### *On the Diseases occasioned by Distortion of the Spinal Column.*

CASE I.—Jan. 31, 1821.—Master George Andrews, of St. Vincent's-place, City-road, aged 13 years, of a florid countenance and fair complexion, leans towards the left in every posture. The leg and thigh of that side are nearly two inches shorter than their fellows. Eight years ago he was accidentally struck with a stone on the false ribs of the same side, at a considerable distance from the spine. He was much hurt, and being unable, from the anguish of the bruise, to raise himself in bed the following morning, a medical gentleman was consulted, who advised an embrocation to be applied. The pains were relieved by it for a few days, but soon returned with equal violence, and extended from the part afflicted to the bottom of the foot. They have continued to the present time with little variation or abatement. He has lately been subject to frequent fits of numbness; they always begin in the bruised part, and proceed from it along the same side to the sole of the foot. Many different remedies have been tried for their removal, but to no good purpose. About eight months after the accident, one of the lower dorsal vertebræ was discovered to project a little. The eminence was at first scarcely perceptible, but increased gradually; and, others following, the swelling has been enlarging ever since. He has for some time been quite well in health, though he is soon fatigued, and easily loses his breath. The heart always palpitates violently, and the apex beats regularly under the third rib. He sleeps well; appetite is good; urine and alvine motions natural. He has a very aged appearance.

On viewing him naked, I found the heads of eight of the upper dorsal vertebræ too prominent, and gradually rising like an inclined plane towards the hump. The remainder, with the connected ribs, had formed a large elevated swelling, inclining towards the right, seven and a half inches long, six and three-quarters broad, and three inches high in the part most raised. Three of the upper lumbar vertebræ assist in forming the lower part of the deformity. The ribs, at their sternal attachments, were very irregularly fixed, and huddled together, especially on the left side, so as to contract the chest in front, and give it a disagreeable appearance. The margin of the ribs on the same side nearly touch the pelvis. Some notion may be formed of the severity of the blow, from its having produced a depression in the part struck, which was very conspicuous in the first model, and not entirely effaced in the last. He is four feet in height, and measures twenty-eight inches round his body at the middle of the hump. His back, from the left between the buttocks to the nape of the neck, measures seventeen and a half inches. The affected limb, when stretched out, becomes numb for a short time, and afterwards prickles, first in one part and then in another.

*Description of Plate I.*—A. Top of the right humerus. B. Ditto left ditto. C. Crista ilei. D. Crease in the skin. E. First dorsal vertebra. F. Division of the nates. G. Impression of the stone. H. Top of the gibbosity. I. Bottom of ditto.

April 10th.—His spine is elongated more than two inches, and the height of the hump is reduced from three inches to one and a half. Both legs are become nearly equal in length, and the hollow on the left side is almost obliterated. The chest is greatly improved in its external form and appearance. His heart beats much less, and has sunk nearly into its proper place. The health is very good, and his countenance has become juvenile.

Length of the hump, at first 7½ inches, is now reduced to 4½; breadth, at first 6¾, is now four inches.

August 10th, 1823.—A gradual detumescence has been observable in the swelling ever since the last report. What remains of it is only perceptible on viewing the naked back; it cannot be discovered through a tight waistcoat. The heart has entirely recovered its proper place in the chest, and his feet are exactly of the same length. He has grown full ten inches since he first lay down, is much plumper, and in excellent health. The circumference of his waist is at this time only twenty inches, though it was full twenty-eight immediately before the treatment began.

Nov. 29th, 1823.—The patient has enjoyed uniform good health ever since the process commenced; he walks quite erect, is very grateful for the recovery of his figure, and the opportunity now afforded him of filling an useful station in society.

*Description of Plate II.*—A. Top of the right shoulder. B. Ditto left ditto. C. Crista ilei. D. Site of the former crease in the skin. E. First dorsal vertebra. F. Division of the nates. G. Impression of the stone.

Many contradictory opinions have been entertained of the nature and causes of spinal distortions. Some of these it may be proper to examine with all the attention which is due to them, while it would only be waste of time to revive such as have already sunk into deserved oblivion.

My present remarks are only applicable to spinal diseases of slow formation, and of a chronic character. They are not meant to include such as originate in hurts, blows, or falls; because, although both have generally been treated indiscriminately, they differ essentially from one another, and should never be confounded.

By carefully distinguishing and keeping them separate, we shall be led to entertain more clear ideas, and to institute a more rational method of treatment. Dislocations occasioned by external violence excite inflammatory symptoms: such as arise from the imperceptible operation of constitutional causes do not produce any increased action, and are seldom accompanied with pain. The relaxed ligaments, being unable to preserve the parts in close connexion, suffer them to gape and recede.

Having, as I conceive, pointed out a distinction of great practical utility, I proceed to observe, that the latter variety of the malady is frequently induced by encouraging awkward habits, and adopting strained positions. During these dangerous indulgences, the spinal column assumes a forced arrangement. The intervertebral substance is compressed in one part, and spread out in the opposite direction. Where this disposition is only temporary, and seldom repeated, the organs implicated recover their natural conformation, and the intervertebral structure is entirely restored by its inherent elasticity. Every time that we bend the back, several vertebral joints are engaged in the manner described, and regain their former condition the moment a different posture is adopted. When particular attitudes are long continued, and frequently repeated, the vertebral joints become at length disposed to give way, and accommodate themselves to the new posture. This inclination is no sooner formed than the vertebræ are observed to move; they afterwards project, and press upon the contiguous membranes; these gradually recede, and consequently stretch. The opposite fibres contract, and in process of time show some resistance to the returning vertebræ. A very dangerous predisposition is now acquired, which can only be counteracted by prompt and efficacious measures. Should these be neglected at this critical juncture, distortion will assuredly supervene. The disposition being once acquired, some of the vertebræ begin to vacillate; they shortly afterward project, and press upon the contiguous fibrous structure; it gradually gives way to the force applied, and consequently stretches; the opposite membranes contract, and oppose some resistance to the vertebræ whenever they are inclined to return to their former situations. The gibbosity of Master George Andrews originated, I conceive, in this way. Being severely hurt in his side by a stone, he, to mitigate the pain, unconsciously bent towards the ailing part. Had he adopted the attitude occasionally, and only for a short time together, his back would probably have maintained its erect figure. Unfortunately the violence and obstinacy of his sufferings led him to lean considerably at all times: the intervertebral structure, in consequence of this habit, became permanently condensed on that side, and equally expanded on the other. The yellow ligaments, which are strong and flexible, soon accommodated themselves to the new posture. The capsular ligaments, and other articulating fibres, stretched gradually in one direction, and contracted equally in another. As soon as these several organs had suited themselves to this unnatural attitude, the deformity began. However slight it might be in the first instance, yet, having once formed, the swelling continued to increase, till, in the space of eight years, it arrived at its present magnitude.

Such was the origin, progress, and termination of this formidable gibbosity. It commenced, as I conceive, in the fibrous texture of the different spinal joints, which, by slowly giving way to the distending force, suffered all the vertebræ of the back and loins to slide out of their natural situations.

Lest it should be concluded, from the preceding narrative, that I am desirous to place all spinal diseases in the same structure, I wish it to be distinctly understood that I never for a moment harboured such an untenable opinion. According to my experience, they sometimes originate in the vertebræ, occasionally in the interposed cartilages, but most commonly in the articular ligaments. It is to the last variety that my remarks will be chiefly directed.

CASE II.—April 3, 1821.—Miss Sarah Sophia Tarrant, of Newington Green, aged nine years, has always been very delicate. It is believed that she was born under the seventh month, but was remarkable for the symmetry of her person and the beauty of her features. Her countenance is dull, sallow, and contracted; the eye is languid, and the face has a haggard and antiquated look. She has a deep and most distressing cough, which returns in fits and at short intervals. Such is its violence, that at every attack the attendants are alarmed with the apprehension of its proving fatal. The dorsal vertebræ rise gradually from the neck, and all the ribs are more or less displaced; the three inferior ones are elevated, with two of the superior lumbar vertebræ, into a large round swelling. Six ribs on the right, and five on the left, swell out with them to assist in making the protuberance. The chest is much contracted in front and behind.

The complaint was occasioned by falling upon the ice in January, was first discovered on the 25th of February, 1820, and had only just commenced: it has been gradually increasing ever since. In the previous autumn she often felt pain in her back after stooping, but no distortion was perceptible till the time mentioned. She measures three feet six inches. Her parents are of opinion that she has grown nothing for the last year and half. Appetite is remarkable, being equal to that of two healthy adults, but her food never seems to impart any nutriment. Bowels are obstinately constipated: the motions have a strong and peculiar odour, something like the washings of a foul gun; they are generally hard, and in some parts clay-coloured, in others have the appearance of pickled walnuts. She is subject to very severe and most distressing pains in her legs and thighs, which are often cold and numb.

May 10th, 1822.—Her spirits are much more lively; she is no longer fretful, and the appearance of the countenance is greatly improved. She has got flesh and weight since she began the treatment. Appetite is natural; her motions are good; she is no longer subject to hasty and irresistible calls to make water. The heart, which beat forcibly, and much below the natural situation, has risen to its proper place; its strokes are no longer violent or particularly strong. The cough is much relieved, both in severity and violence. The protuberance is greatly reduced: nine of the eleven ribs have nearly returned to their proper places; two on the right side remain something displaced. She has increased in height two inches.

June 25th, 1822.—She is always cheerful, is much improved in her general appearance, and has an open countenance. The cough has entirely left her. Her chest and loins are much broader, and finely turned. Appetite is regular and good, but not extraordinary; she lives upon fresh animal food for dinner, with a suitable proportion of vegetable matter, and the diet agrees with her. The ribs on the left side have been some time replaced; the eighth and ninth on the right side are slightly elevated. All the vertebræ, except the eleventh and twelfth dorsal and first lumbar, have regained their natural stations, and these are not more than a quarter of an inch too high.

October 10th, 1823.—The recumbent posture has been entirely observed, and her health has been uniformly good, ever since the last report. The swelling is wholly removed, except in the three vertebræ formerly mentioned, which are still slightly protuberant. The ribs have all of them been some time reinstated. She now measures in length four feet four inches.

Dec. 5th, 1823.—Miss S. S. Tarrant gets up every day, and walks about for some time with great ease to herself. The health, complexion, and general appearance, continue unimpaired.*

In this young sufferer, the improvement in health constitutes a feature of the case as striking and important as the recovery of her figure. One of the most eminent and experienced of the metropolitan faculty had declared, only a few days before I was first consulted, that she would not survive three months. An harassing cough, a wasting hectic, and other alarming symptoms, were consuming her strength, which a voracious appetite was unable to counteract. She was in such an alarming situation at my first visit, that I submitted my opinion to the parents in a written report; the contents of which met the approbation of their medical attendant, a gentleman of great professional experience and respectability. The report was couched in the following terms:

"Feb. 19th, 1821.—On examination this morning, I found three of the lower dorsal and two of the upper lumbar vertebræ partially displaced in the person of Miss S. S. Tarrant: one had risen considerably above the rest, and its spinal process stood out prominently and alone. The ribs on both sides, by their firm attachments to the vertebræ, are forced almost directly backwards, and, together with these protuberant vertebræ, constitute the large roundish swelling which is so conspicuously placed below the shoulder-blades. In front are several marked depressions, or lines, on each side, extending from the sternum to the back: they were produced by the posterior or outward

* The insertion of the engravings in illustration of this case is postponed till next month, in consequence of our having already two copper-plates in the present Number, to represent the preceding cases.

spinal curve having drawn the lower ribs along with it. In consequence of these deviations, the chest is peaked and contracted. The heart beats with unusual force, to drive the blood through the displaced pulmonary vessels and aorta; a displacement which is the unavoidable consequence of their being firmly bound internally to the bent spine. From the derangement of the spine and chest, the heart, lungs, stomach, and liver, must necessarily be squeezed and dangerously compressed, so as to impede and disturb their important functions. The patient has from these circumstances been reduced to infirm and delicate health, and become predisposed to pulmonary consumption. This very dangerous state can only be removed by restoring the back and chest to their natural form, that the spinal nerves and internal organs may be reinstated in their natural abilities."

I had the satisfaction to observe that, under the treatment, she advanced rapidly, and quickly succeeded to a good state of general health. Nor has it, during the whole period, suffered the slightest interruption, either from constant recumbency or the other means employed for her cure. She has through the whole confinement been cheerful and happy, from a strong conviction in her own mind that the plan she was pursuing would ultimately restore her to the full possession and enjoyment of all her faculties. I have the gratification to add, that her patience and most sanguine anticipations have been amply rewarded.

The busts of both patients were modelled from nature by Mr. Mazzoni. That the engravings might exhibit a faithful picture of the parts intended to be represented, the corresponding drawings are taken in the same position of the casts.

Having described the commencement, progress, and termination, of two instances of spinal deformity, in which, from length of time, the parts had grown firm, rigid, and nearly immovable, I proceed to relate another example of extraordinary distortion, to show with what ease the spinal column can, in recent cases, be restored to its natural arrangement.

The good effects of the usual treatment were on this occasion so immediate and striking, that I should not have ventured to publish the case, had I not been permitted by the family of the young lady to disclose her name and residence. Thus fortified, I have not hesitated to bring it forward; nor to add, that I have been equally successful with several other patients, who applied for my assistance at an early period, and under the same favourable circumstances.*

---

* We regret that we are obliged to postpone till next month the continuation of Dr. HARRISON's observations, wherein he proceeds to detail the method of treatment he adopts.—EDITORS.

## Figures and Tables

**Figure f1:**
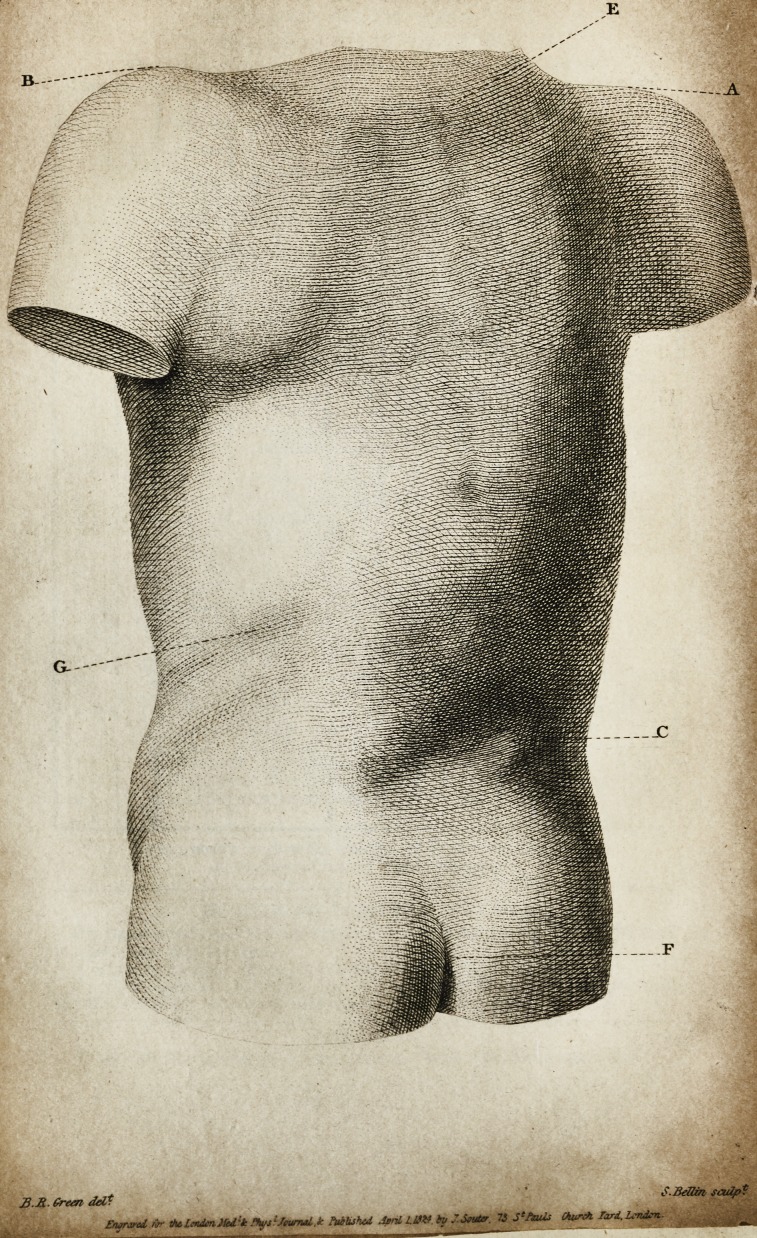


**Figure f2:**